# Burden of Influenza-Associated Respiratory Hospitalizations, Vietnam, 2014–2016

**DOI:** 10.3201/eid2710.204765

**Published:** 2021-10

**Authors:** Nguyen Cong Khanh, Ashley L. Fowlkes, Ngu Duy Nghia, Tran Nhu Duong, Ngo Huy Tu, Tran Anh Tu, Jeffrey W. McFarland, Thoa Thi Minh Nguyen, Nga Thu Ha, Philip L. Gould, Pham Ngoc Thanh, Nguyen Thi Huyen Trang, Vien Quang Mai, Phuc Nguyen Thi, Satoko Otsu, Eduardo Azziz-Baumgartner, Dang Duc Anh, A. Danielle Iuliano

**Affiliations:** National Institute of Hygiene and Epidemiology, Hanoi, Vietnam (K.C. Nguyen, N.D. Ngu, D.N. Tran, T.H. Ngo, T.A. Tran, A.D. Dang);; US Centers for Disease Control and Prevention, Atlanta, Georgia, USA (A.L. Fowlkes, J.W. McFarland, E. Azziz-Baumgartner, A.D. Iuliano);; US Centers for Disease Control and Prevention, Hanoi, Vietnam (T.T.M. Nguyen, N.T. Ha, P.L. Gould);; Tay Nguyen Institute of Hygiene and Epidemiology, Buon Ma Thuot, Vietnam (T.N. Pham);; Pasteur Institute Ho Chi Minh, Ho Chi Minh City, Vietnam (T.T.H. Nguyen); Pasteur Institute Nha Trang, Nha Trang, Vietnam (M.Q. Vien);; World Health Organization Vietnam Country Office, Hanoi (N.T. Phuc, S. Otsu)

**Keywords:** influenza, burden, vaccines, incidence, hospitalization, viruses, respiratory infections, Vietnam

## Abstract

Influenza burden estimates are essential to informing prevention and control policies. To complement recent influenza vaccine production capacity in Vietnam, we used acute respiratory infection (ARI) hospitalization data, severe acute respiratory infection (SARI) surveillance data, and provincial population data from 4 provinces representing Vietnam’s major regions during 2014–2016 to calculate provincial and national influenza-associated ARI and SARI hospitalization rates. We determined the proportion of ARI admissions meeting the World Health Organization SARI case definition through medical record review. The mean influenza-associated hospitalization rates per 100,000 population were 218 (95% uncertainty interval [UI] 197–238) for ARI and 134 (95% UI 119–149) for SARI. Influenza-associated SARI hospitalization rates per 100,000 population were highest among children <5 years of age (1,123; 95% UI 946–1,301) and adults >65 years of age (207; 95% UI 186–227), underscoring the need for prevention and control measures, such as vaccination, in these at-risk populations.

Annual circulation of influenza viruses causes substantial disease and death worldwide, disproportionately affecting young children and older adults (*1*,*2*). Globally, influenza causes ≈9.4 million hospitalizations each year (*3*) but remains an underrecognized contributor to hospitalizations in many countries (*4*). In 2014, the World Health Organization (WHO) published a manual to guide countries to estimate influenza disease burden using influenza surveillance data (*5*–*10*). Such estimates have been instrumental in demonstrating influenza as a common cause of hospitalization in tropical and low- and middle-income countries (*11*), including Southeast Asia countries (*8*,*10*,*12*), and have provided a platform for evaluating the cost-effectiveness of influenza vaccines. Country-specific burden estimates can inform decisions to invest resources in influenza prevention and control programs (*4*,*13*), whereas studies from other countries may not be sufficiently compelling among national leaders to guide policy or investments (*14*).

During the past 40 years, Vietnam has experienced rapid economic growth, shifting from a low-income to a lower middle-income classification (*15*). Correspondingly, government spending on national health programs has increased, including efforts to address public health threats from human and zoonotic influenza (*14*). Vietnam has had sustained influenza surveillance programs since 2006, generating data about influenza virus circulation through influenza-like illness (ILI) and severe acute respiratory infection (SARI) surveillance. In 2019, the Institute of Vaccines and Medical Biologicals successfully licensed the first locally manufactured seasonal influenza vaccine (*16*). To sustain Vietnam’s influenza prevention and control programs, information about the annual disease burden and value of averting costly hospitalizations is useful. Building on the established SARI sentinel surveillance, we conducted a hospital admission survey (HAS) to estimate the national disease burden of influenza-associated hospitalization.

## Methods

Vietnam’s 63 provinces are divided into the north, central, highlands, and south major health regions. The healthcare system includes both public and private hospitals, but public hospitals are most commonly used (*17*). In 2011, the Vietnam Ministry of Health began adopting electronic medical records (EMRs) in all public hospitals (*18*,*19*). For the HAS, we selected 1 province per region that had all public hospitals using EMRs, a nearby provincial-level hospital enrolled in SARI surveillance using trained surveillance officers, and no private hospitals routinely admitting acute respiratory infection (ARI) patients.

To estimate the influenza-associated respiratory hospitalization rate in Vietnam for comparison with other countries, we combined existing virologic data from SARI sentinel surveillance (*20*), EMR data, and a medical record review of a random set of ARI hospitalizations (*5*). Influenza burden data were interpreted in conjunction with influenza seasonality to inform vaccine formulation considerations for maximizing population benefit.

### Data Sources

#### ARI Hospitalizations

We defined an ARI hospitalization as a hospitalization with an admission code from the International Classification of Diseases, 10th Revision (ICD-10), for either acute upper respiratory infection (J06) or codes to approximate SARI (influenza, J09–11; pneumonia, J12–18; or other acute lower respiratory infections, J20–J22) (*5*). An overnight stay was not required because of variability in thresholds for admission, cultural practice of taking the most severely ill patients home, minimum availability of precise admission and discharge times, and possible patient transfer. Using the EMR system, public hospitals provided a list of patients with an ARI admission code during 2014–2016 with the following data: admission and discharge ICD-10 codes; hospital admission and discharge dates; patient residential province, age, and sex; and outcome of hospitalization at discharge.

#### Medical Record Review to Identify SARI Proportion

From ARI hospitalizations for each hospital, a random selection of medical records was reviewed by health officers and clinical data collected to evaluate both the 2011 and 2014 WHO SARI case definitions. The 2011 definition was sudden onset of fever >38°C, with cough or sore throat, and with shortness of breath or difficulty breathing, and illness requiring hospitalization; the revised 2014 definition was temperature of >38°C or history of fever, cough of duration <10 days, and illness requiring hospitalization. We assumed that 50% of ARI hospitalizations would meet SARI case criteria and assigned 80 records per hospital to reach a sufficient sample size with a 5% margin of error.

#### Influenza Test Results from SARI Sentinel Surveillance

In 2011, Vietnam initiated SARI surveillance in sentinel hospitals representing the 4 major regions of Vietnam; 6–14 hospitals participated per year. Each week, sentinel hospitals reported the total number of SARI admissions. Surveillance staff collected nasopharyngeal and oropharyngeal swabs from 8 SARI patients per week (protocols varied by hospital and ward). Demographic and clinical information also were collected. The National Influenza Center conducted real-time reverse transcription PCR to detect influenza A (subtypes A(H1N1)pdm09, A/H3, A/H5, and A/H7) and B (*20*). Influenza virologic surveillance data were obtained from all SARI sentinel hospitals participating in surveillance during 2014–2016.

#### Population Data

A national census survey was conducted in 2009 with annual population projections (*21*). To calculate population-based rates of ARI and SARI, we used 2014–2016 provincial and national population projections for 4 age groups: <5 years, 5–49 years, 50–64 years, and >65 years. Evaluation of 14 demographic, health, and healthcare characteristics in each province within the 4 regions demonstrated that provinces selected for the HAS were representative and could be combined for a national estimate of ARI and SARI rates in Vietnam (Appendix).

### Data Analysis

#### ARI and SARI Hospitalizations

We reviewed the reported ARI hospitalizations and excluded patients with non-ARI ICD-10 codes and those residing outside of the province. Eight hospitals in 2014 and 7 in 2015 reported zero or near-zero ARI hospitalizations during the introduction of the EMR system. To compensate for underreporting, we used the individual hospital’s 2016 ARI counts to estimate the expected number of hospitalizations in 2014 and 2015. We further adjusted for patients with missing age information by using the distribution of patients with known ages (Appendix Figure 1). We assessed the percentage of ARI hospitalizations meeting the SARI case definition by province, hospital, year, age group, and ICD-10 code.

#### Estimating the Rate of Influenza-Associated Hospitalizations

We estimated the influenza-attributable proportion of ARI and SARI hospitalizations by using 2014–2016 SARI sentinel surveillance data. We calculated influenza-associated ARI and SARI hospitalization rates by multiplying the age- and month-specific number of hospitalizations and percentage of SARI surveillance patients positive for influenza, then dividing by the age-specific census population estimates for each province. We summed the monthly rate estimates to calculate the annual age-adjusted rates. We calculated the 95% uncertainty intervals (UI) for estimated rates by using 1,000 Monte Carlo simulation iterations, assuming a Poisson distribution for the number of hospitalizations and a binomial distribution for the proportion of SARI patients positive for influenza. We used the same method to calculate influenza-associated SARI hospitalization rates, but first we multiplied ARI hospitalization totals by the percentage of ARI hospitalizations meeting SARI criteria to obtain the age- and month-specific number of influenza-associated SARI hospitalizations. The 95% UIs assumed a binomial distribution for the proportion meeting the SARI case definition. We extrapolated the number of influenza-associated ARI and SARI hospitalizations by multiplying age-specific influenza rates by the provincial census population.

To estimate national rates, we summed age- and month-specific provincial counts of all-cause ARI and SARI and influenza-associated ARI and SARI across the provinces, and then divided the sum by the total population of the 4 provinces. We obtained the 95% UIs by calculating the upper and lower 2.5% percentiles from the distribution of provincial rate estimates.

We used χ^2^ tests of proportions to assess statistical differences and calculate 95% CIs by using the observed data when appropriate. We performed all analyses using SAS 9.4 (SAS Institute, https://www.sas.com).

This activity was reviewed by the US Centers for Disease Control and Prevention and the ethics committee and scientific committee of the National Institute of Hygiene and Epidemiology (Hanoi, Vietnam). The data are considered nonresearch; therefore, Institutional Review Board review was not required.

## Results

### Reported ARI Hospitalizations

**Figure 1:**
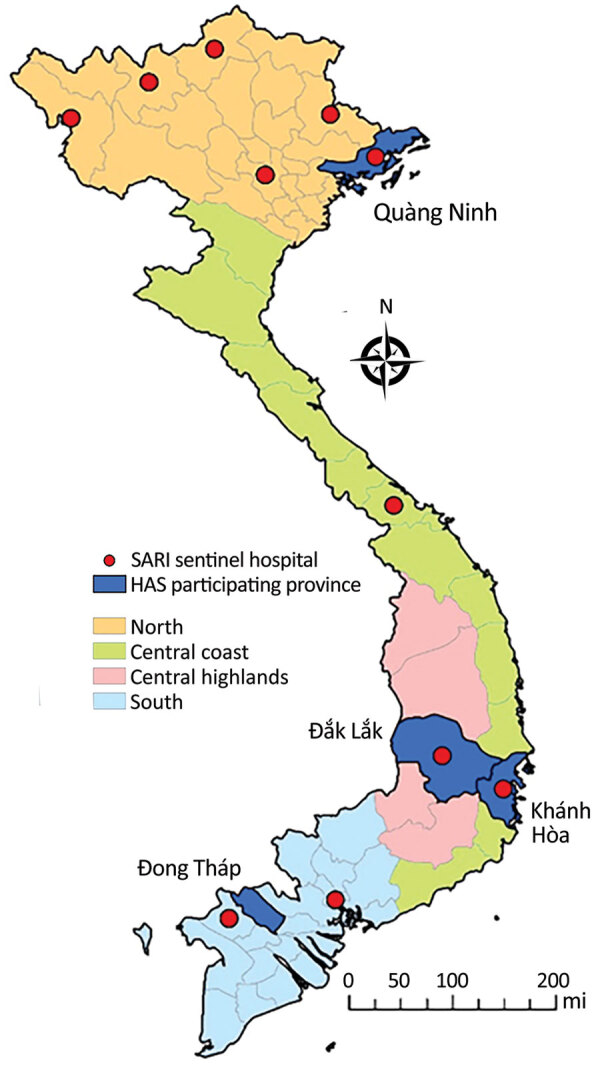
Locations of sentinel hospitals conducting surveillance for severe acute respiratory infection and provinces with all hospitals participating in the hospital admission survey, Vietnam, 2014–2016. HAS, hospital admission survey; SARI, severe acute respiratory infection.

The provinces identified for the HAS included Quang Ninh (14 hospitals) in the north region, Khanh Hoa (10 hospitals) in the central region, Dak Lak (15 hospitals) in the highlands region, and Dong Thap (12 hospitals) in the south region (Figure 1). During January 2014–December 2016, a total of 220,217 ARI hospitalizations were reported, excluding 2,781 patients living outside of the province and 4,960 patients who did not have qualifying ARI ICD-10 codes. We identified 8 hospitals with clear underreporting during EMR implementation in 2014 and 2015 and imputed the expected number of ARI hospitalizations by using the 2016 percentage distribution of patient counts across hospitals, giving an overall estimated 4.1% underreporting of ARI hospitalizations and an adjusted total of 229,144 ARI hospitalizations included in analysis.

Among patients with known age, 61% were <5 years, 15% were 5–49 years, 7.4% were 50–64 years, and 17% were >65 years (Table 1). The median length of hospitalization was 6 days (interquartile range 4–6 days). Among 180,316 ARI hospitalizations with a known discharge disposition, 482 (0.3%) patients died and 4,376 (2.4%) were sent home as too severely ill to be cured.

**Table 1:** Percentages of ARI hospitalizations that met SARI case criteria from medical chart review, by age group, qualifying admission code, and province, Vietnam, 2014–2016*

Category	No. (%) hospitalization		
	All ARI hospitalizations†	Medical record reviewed	Met SARI criteria‡
Overall	220,217 (100)	3,626 (100)	2,205 (60.8)
Age group, y§			
<5	132,076 (61.0)	1,934 (53.4)	1,443 (74.6)
5–49	32,112 (14.8)	716 (19.8)	421 (58.8)
50–64	16,030 (7.4)	324 (8.9)	123 (38.0)
>65	36,329 (16.8)	647 (17.9)	217 (33.5)
Diagnosis, ICD-10 code			
Acute upper respiratory infections, J06	37,957 (17.2)	459 (12.7)	325 (70.8)
Influenza	4,278 (1.9)	82 (2.3)	30 (36.6)
Influenza caused by other identified influenza virus, J10	963 (0.4)	36 (1.0)	9 (25.0)
Influenza caused by unidentified influenza virus, J11	3,315 (1.5)	46 (1.3)	21 (45.7)
Pneumonia	117,890 (53.5)	1,946 (53.7)	271 (13.9)
Viral pneumonia, not elsewhere classified, J12	470 (0.2)	10 (0.3)	7 (70.0)
Pneumonia caused by *Streptococcus pneumoniae*, J13	82 (0)	9 (0.2)	7 (77.8)
Pneumonia caused by *Hemophilus influenzae*, J14	51 (0)	0 (0)	0 (0)
Bacterial pneumonia, not elsewhere classified, J15	22,252 (10.1)	399 (11.0)	257 (64.4)
Pneumonia caused by other infectious organisms, J16	10,629 (4.8)	40 (1.1)	28 (70.0)
Pneumonia in diseases classified elsewhere, J17	65 (0)	3 (0.1)	3 (100.0)
Pneumonia, unspecified organism, J18	84,341 (38.3)	1,485 (41.0)	970 (65.3)
Other acute lower respiratory infections¶	60,092 (27.3)	1139 (31.4)	578 (50.7)
Acute bronchitis, J20	55,492 (25.2)	1,096 (30.2)	547 (49.9)
Acute bronchiolitis, J21	4,563 (2.1)	43 (1.2)	31 (72.1)
Unspecified acute lower respiratory infection, J22	37 (0)	0 (0)	0 (0)
Province			
Dak Lak, 15 hospitals	60,805 (27.6)	853 (23.5)	567 (66.5)
Dong Thap, 12 hospitals	67,746 (30.8)	828 (22.8)	495 (59.8)
Khanh Hoa, 10 hospitals	56,187 (25.5)	854 (23.6)	522 (61.1)
Quang Ninh, 14 hospitals	35,479 (16.1)	1,091 (30.1)	612 (56.1)

*ARI, acute respiratory infection; ICD-10, International Classification of Diseases, 10th Revision; SARI, severe acute respiratory infection. †Percentages show the overall proportion of patients in the listed category (column percentage). ‡Percentages show the proportion of patients with illness meeting SARI criteria (row percentage), defined as temperature of >38°C or history of fever, cough of duration <10 d, and illness requiring hospitalization; all proportions varied substantially within categories. §Age data were missing for 3,670 ARI and 5 patients with medical record review. ¶Admission ICD codes J14 and J22 were not identified among reviewed patient records; J09 was not identified among any patients.

### Estimated SARI Hospitalizations

Of 3,626 medical record reviews from ARI hospitalizations, 61% met the SARI case definition, whereas 27% did not have documented fever, 8% did not have cough, and 4% had neither. SARI accounted for 75% of ARI hospitalizations among children <5 years of age, compared with patients 5–49 years of age (59%), 50–64 years of age (38%), and >65 years of age (34%) (p<0.001) (Table 1). The ICD-10 codes most commonly listed for patients with illness meeting SARI criteria were acute bronchiolitis (J21 [72%]) and acute upper respiratory tract infection (J06 [71%]) among codes reported for >10 patients. Influenza codes were reported infrequently, and significantly less frequently among patients with illness meeting the SARI criteria (37%) compared with other diagnoses (p<0.001) (Table 1).

### Influenza Detection

The SARI sentinel surveillance collected specimens from 6,647 patients. Influenza detection varied significantly by age (p<0.001), and was less frequent among children <5 years of age (13% [95% CI 12%–14%]) and patients >65 years of age (16% [95% CI 14%–18%]) compared with patients 5–49 years of age (23% [95% CI 21%–24%]) and patients 50–64 years of age (23% [95% CI 20%–25%]). The age-weighted proportion of samples testing positive for influenza was 22% (95% CI 21%–23%), varying by year (20% in 2014, 18% in 2015, and 23% in 2016). Influenza detections did not vary substantially by region (Appendix Figure 2); therefore, we combined data to allow stratification by age and month.

**Figure 2:**
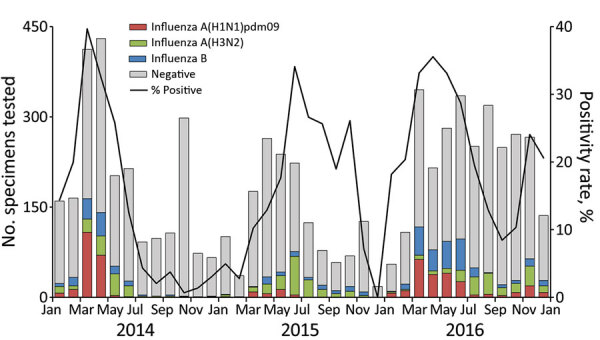
Number of specimens tested and percentage testing positive for influenza viruses among severe acute respiratory infection sentinel surveillance system patients, by month, Vietnam, 2014–2016.

Across all years, ≈74.4% of influenza detections occurred during March–July (Figure 2). Influenza A viruses predominated in 2014 and 2015; subtype H1N1 made up 44% of viruses detected in 2014 and subtype H3N2 65% of viruses detected in 2015. In 2016, influenza viruses cocirculated; 36% were A(H1N1), 28% A(H3N2), and 35% B, but H1N1 and B co-circulated during March–June, whereas most H3N2 detections occurred during June–November (Figure 2).

### Rates of Influenza-Associated ARI and SARI Hospitalizations

#### Provincial Rates of Influenza-Associated ARI and SARI Hospitalizations

During 2014–2016, the mean annual influenza-associated ARI hospitalization rates ranged by province from 183 (95% UI 159–206) to 284 (95% UI 256–312) per 100,000 population (Table 2). The provincial influenza-associated ARI hospitalization rates were lower in 2014, when influenza viruses were primarily detected during January–June, compared with 2016, when influenza was detected throughout the year and all 3 viruses circulated.

**Table 2:** Provincial estimates of influenza-associated ARI and SARI hospitalizations and rates for all ages, Vietnam, 2014–2016*

Year and province	ARI Hospitalizations				Estimated SARI Hospitalizations		
	No.	No. (95% UI)			No. (95% UI)		
		Rate	Influenza-associated rate		No.	Rate	Influenza-associated rate
Mean annual							
Dak Lak	20,746	1,107 (1,091–1,122)	183 (159–206)		14,093 (13,861–14,308)	752 (739–763)	121 (104–139)
Dong Thap	22,582	1,314 (1,297–1,330)	224 (200–247)		13,561 (13,352–13,772)	789 (777–801)	133 (118–148)
Khanh Hoa	20,433	1,665 (1,645–1,686)	284 (256–312)		12,760 (12,558–12,957)	1,040 (1,023–1,056)	177 (157–197)
Quang Ninh	12,620	1,030 (1,011–1,046)	197 (180–215)		7,423 (7,280–7,553)	606 (594–616)	113 (103–124)
2014							
Dak Lak	18,592	1,004 (988–1,021)	103 (90–116)		12,711 (12,487–12,929)	687 (675–698)	66 (57–75)
Dong Thap	22,188	1,299 (1,281–1,319)	151 (134–171)		13,403 (13,178–13,647)	785 (772–799)	90 (80–102)
Khanh Hoa	18,788	1,546 (1,523–1,570)	195 (174–219)		11,793 (11,578–12,003)	971 (953–988)	119 (105–135)
Quang Ninh	11,171	921 (902–938)	137 (122–153)		6,877 (6,714–7,037)	567 (553–580)	83 (73–94)
2015							
Dak Lak	19,605	1,046 (1,030–1,061)	192 (145–243)		13,346 (13,116–13,572)	712 (700–724)	133 (96–173)
Dong Thap	20,655	1,202 (1,185–1,219)	219 (171–269)		12,384 (12,168–12,597)	720 (708–733)	131 (100–163)
Khanh Hoa	20,919	1,705 (1,682–1,728)	292 (239–344)		13,055 (12,837–13,273)	1,064 (1,046–1,082)	184 (148–220)
Quang Ninh	11,216	915 (893–935)	161 (136–188)		6,614 (6,450–6,768)	540 (526–552)	93 (76–111)
2016							
Dak Lak	24,041	1,266 (1249–1,283)	251 (228–272)		16,220 (15,974–16,467)	854 (841–867)	164 (149–178)
Dong Thap	24,903	1,440 (1422–1,459)	299 (273–324)		14,896 (14,660–15,134)	861 (847–875)	177 (161–193)
Khanh Hoa	21,593	1,743 (1720–1,768)	363 (332–397)		13,434 (13,196–13,651)	1,084 (1,065–1,102)	226 (205–250)
Quang Ninh	15,473	1,251 (1227–1,273)	291 (264–321)		8,776 (8,579–8,956)	709 (693–724)	162 (149–177)

*Rates are given per 100,000 population and age-adjusted. Rates were calculated regionally using reported ARI hospitalizations multiplied by the percentage of patients meeting the SARI criteria upon medical chart review. Regional hospitalization counts were multiplied by the national percentage of specimens testing influenza-positive from SARI sentinel surveillance, adjusted by age and month, and then divided by the combined population of the regions. ARI, acute respiratory infection; SARI, severe acute respiratory infection. UI, uncertainty interval.

#### National Rates of ARI Hospitalizations and Influenza-Associated ARI Hospitalizations

Across the 4 provinces, the mean annual ARI hospitalization rate was 1,263 (95% UI 1,248–1,278)/100,000 population and the influenza-associated ARI hospitalization rate was 218 (95% UI 197–238)/100,000 population (Table 3). The age-adjusted rate of influenza-associated ARI was higher in 2016 (295 [95% UI 273–318]/100,000 population) compared with 2014 (142 [95% UI 128–157]/100,000 population). After extrapolating provincial rates to Vietnam’s population of ≈93 million during 2014–2016, we estimated that 129,019 influenza-associated ARI hospitalizations occurred in 2014, 195,795 in 2015, and 273,357 in 2016. Using the extrapolated counts, we found that the mean influenza-associated ARI hospitalizations during April–September, when Southern Hemisphere influenza vaccines typically are available, was 116,324 compared with 69,493 during October–March, when Northern Hemisphere influenza vaccines typically are available (Appendix Figure 3).

**Table 3:** National influenza-associated acute respiratory infection (ARI) and severe acute respiratory infection (SARI) hospitalization and rate estimates, Vietnam, 2014–2016

Year and age group, y	ARI hospitalizations, no. (95% UI)				SARI hospitalizations, no. (95% UI)		
	Mean regional rate	Mean regional influenza-associated rate	Extrapolated national influenza-associated cases		Mean regional rate	Mean regional influenza-associated rate	Extrapolated national influenza-associated cases
Mean annual							
All	1,263 (1,248–1,278)	218 (197–238)	199,368 (180,126–217,895)		791 (779–803)	134 (119–149)	122,832 (109,263–136,377)
<5	9,530 (9,372–9,693)	1,508 (1,272–1,745)	114,338 (96,479–132,357)		7,103 (6,966–7,236)	1,123 (946–1,301)	85,191 (71,749–98,623)
5–49	257 (253–260)	56 (53–60)	36,738 (34,767–39,225)		146 (143–148)	32 (30–34)	20,876 (19,660–22,312)
50–64	690 (658–717)	155 (132–181)	20,150 (17,093–23,480)		272 (253–291)	61 (51–72)	7912 (6,654–9,354)
>65	3,776 (3,730–3,820)	600 (545–656)	34,527 (31,367–37,757)		1,301 (1,275–1,326)	207 (186–227)	11,883 (10,729–13,064)
2014							
All	1,181 (1,167–1,196)	142 (128–157)	129,019 (116,354–142,617)		748 (737–759)	87 (77–97)	78,905 (70,212–88,261)
<5	9,201 (9,051–9,358)	942 (792–1105)	71,035 (59,718–83,357)		6,843 (6,714–6,970)	698 (587–818)	52,663 (44,230–61,689)
5–49	223 (218–228)	41 (38–44)	26,821 (24,830–28,928)		127 (123–131)	24 (22–26)	15,326 (14,149–16,601)
50–64	606 (582–629)	91 (74–113)	11,341 (9,219–13,987)		242 (226–258)	37 (29–46)	4,553 (3,587–5,681)
>65	3,446 (3,380–3,519)	427 (355–504)	24,073 (20,032–28,429)		1,195 (1,154–1,236)	147 (120–177)	8,307 (6,781–9,969)
2015							
All	1,197 (1,182–1,212)	214 (175–254)	195,795 (160,673–232,667)		751 (739–762)	134 (107–164)	123,124 (98,168–149,830)
<5	9,114 (8,961–9,270)	1,642 (1,183–2,115)	124,690 (89,882–160,624)		6,798 (6,666–6,927)	1,226 (886–1,583)	93,111 (67,328–120,261)
5–49	233 (228–238)	40 (36–46)	26,219 (23,210–30,057)		131 (128–135)	23 (20–26)	14,803 (13,087–17,042)
50–64	655 (619–686)	128 (96–160)	16,572 (12,422–20,720)		260 (239–282)	50 (37–64)	6,514 (4,811–8,362)
>65	3,594 (3,524–3,661)	613 (506–722)	35,180 (29,065–41,434)		1,233 (1,195–1,268)	210 (173–250)	12,073 (9916–14,337)
2016							
All	1,409 (1,391–1,426)	295 (273–318)	273,357 (252,921–294,062)		874 (860–887)	180 (165–195)	166,505 (152,831–180,715)
<5	10,271 (10,095–10,450)	1,935 (1,705–2,185)	147,341 (129,794–166,383)		7,664 (7,507–7,810)	1,443 (1,267–1,625)	109,840 (96,439–123,710)
5–49	314 (308–319)	87 (81–94)	57,237 (53,188–61,587)		178 (174–182)	50 (46–54)	32,533 (30,011–35,101)
50–64	799 (763–831)	240 (196–283)	32,395 (26,510–38,173)		312 (291–332)	93 (77–111)	12,613 (10,375–15,037)
>65	4,264 (4,193–4,332)	752 (657–867)	44,234 (38,629–51,022)		1,468 (1,426–1,506)	259 (225–299)	15,238 (13,251–17,595)

*Rates are given per 100,000 population and age-adjusted unless shown for a single age group. Rates were calculated regionally using reported ARI hospitalizations multiplied by the percentage of patients meeting the SARI criteria upon medical chart review. Regional hospitalization counts were multiplied by the national percentage of specimens testing influenza-positive from SARI sentinel surveillance, adjusted by age and month, and then divided by the combined population of the regions. ARI, acute respiratory infection; SARI, severe acute respiratory infection; UI, uncertainty interval.

#### National Rates of Influenza-Associated SARI

We estimated age-adjusted influenza-associated SARI rates per 100,000 population of 87 in 2014, 134 in 2015, and 180 in 2016. The mean age-adjusted annual rate was 134 (95% UI 119–149)/100,000 population. Children <5 years of age had the highest rates of influenza-associated SARI (1,123 [95% UI 946–1,301]/100,000 population), followed by adults aged >65 years (207 [95% UI 186–227]/100,000 population).

## Discussion

Using HAS methodology in 4 of Vietnam’s provinces during 2014–2016, we estimated influenza virus infections were associated with 123,000–200,000 respiratory hospitalizations each year and demonstrated influenza as a common cause of hospitalization in young children and older adults. We estimated the influenza-associated hospitalization rate using both an expanded set of ICD-10 codes to define our ARI case definition (218/100,000 population) and a medical chart review to establish rates with the commonly used SARI case definition (134/100,000 population). Among age groups specifically recommended by WHO for vaccination (*22*), we estimated higher rates of hospitalization among children <5 years of age (ARI, 1,508/100,000 population; SARI, 1,123/100,000 population) and adults >65 years of age (ARI, 600/100,000 population; SARI, 207/100,000 population). We also found in any year that 74% of influenza detections were identified during March–July, when Southern Hemisphere influenza vaccines typically are available.

In Vietnam, influenza viruses were detected among 18%–23% of SARI patients, a finding that is consistent with assessments of influenza detection among SARI patients conducted in New Zealand and Hong Kong (*23*,*24*) but higher than those reported in systematic reviews that focus specifically on acute lower respiratory tract infections (*2*,*3*). Our influenza-associated SARI hospitalization rates were similar to those found in a study in the Philippines that used comparable methods (*25*) and other studies in Bhutan, Korea, Thailand, and China that reported a comparable percentage of cases positive for influenza (*10*,*26*–*28*). Our estimates among children <5 years of age (1,123/100,000 population) were 5–35 times higher than the other age groups, potentially reflecting differences in healthcare utilization. The government of Vietnam pays all medical costs for children <5 years of age, thus removing important barriers to healthcare access. Conversely, among adults >65 years of age, our estimated influenza-associated SARI hospitalization rate (207/100,000) was lower than those reported from the United States and Hong Kong (*29*,*30*).

Influenza burden estimates specific to Vietnam support local decision making and targeted risk communications for subpopulations at higher risk for influenza-associated complications and clinicians for targeted use of treatment and nonpharmaceutical interventions. In supporting decision-making for governmental leaders, burden estimates can provide evidence to sustain influenza surveillance programs and investments in pandemic planning, influenza treatment, and vaccination programs. The government of Vietnam has supported influenza virus surveillance and supported the development of the first licensed, locally produced influenza vaccine. Vaccination remains the best way to prevent influenza (*29*), and establishing a strong seasonal vaccine program may also be useful in providing the necessary infrastructure required for pandemic vaccines in the event of an emergent influenza virus or other pandemic respiratory viruses, such as severe acute respiratory syndrome coronavirus 2 (*31*). Burden estimates also can help in understanding the potential impact of a possible future vaccination program.

The seasonal distribution of influenza detections suggested circulation in Vietnam occurred primarily during January–July. We demonstrated that 75% of influenza detections were observed during March–July, which was consistent when evaluating all available years of SARI sentinel surveillance data, including data published previously from 2011–2014 (*20*). Nguyen et al. demonstrated variable timing of peaks for influenza A and B viruses; 76% of influenza A viruses were detected in May–October, and little pattern was observed for influenza B viruses (*32*). Our findings are similar to those in neighboring Thailand (*27*) and Cambodia (*33*), suggesting that most influenza circulation occurs during the rainiest months (April–August) (*34*) and that a Southern Hemisphere vaccine formulation is appropriate for use in Vietnam.

To align our work with existing literature and evaluate the broad potential burden of influenza, we studied influenza in patients with illness meeting the SARI and ARI case definitions. Previous studies have demonstrated that the SARI case definition is less sensitive and more specific (*35*), ensuring more efficient use of resources for virologic surveillance; however, the actual influenza hospitalization burden may be underestimated. A more sensitive case definition is needed to encompass the entirety of influenza disease burden. Furthermore, a recent review of influenza-associated hospitalization rates underscored the effect of heterogeneity in methods and case definitions on burden estimates (*36*). A strength of our study is the use of a medical chart review to identify SARI in ≈60% of hospitalized patients with ARI, which is consistent with a previous study evaluating the sensitivity of administrative codes to identify SARI (*37*) and enabled us to provide burden estimates that may contribute to pooled estimates in studies using different case definitions. Additional research is needed to understand the relationship between diagnostic codes indicative of ARI, the SARI case definition, and influenza detection.

The first limitation of our study is that we were only able to perform the HAS in 4 of Vietnam’s 63 provinces. Although our comparison of provincial characteristics demonstrated minimal variation within each region from the HAS referent provinces, we could not account for many characteristics that might lead to differences in influenza hospitalization rates, such as healthcare-seeking behaviors. Second, the EMR transition was delayed in some hospitals during 2014 and 2015, resulting in underestimation of the number of respiratory hospitalizations recorded. We imputed an expected number of hospitalizations for hospitals with known delays and increased the total ARI hospitalization accordingly, but other hospitals may have had unidentified implementation challenges that were not captured. Third, in accordance with reporting procedures in Vietnam, only the primary reason for a hospitalization was listed in the EMR for each patient (*37*). Although this practice serves to indicate the primary reason for a hospitalization, patients with other conditions in conjunction with a respiratory illness may be missed (*38*). Fourth, participation in SARI surveillance was not continuous for all hospitals, potentially underrepresenting the central and highlands regions and resulting in insufficient sample size to calculate both age- and region-specific influenza detection percentage positives. However, we identified minimal variability in region-specific influenza circulation and thus stratified analyses by age only (Appendix).

Our results highlight the burden of influenza-associated hospitalizations in Vietnam during 2014–2016 and underscore the value of country-specific disease burden studies. As Vietnam undertakes the production of influenza vaccine locally, influenza prevention and control investments and well-timed public health interventions such as vaccination campaigns and empiric antiviral use during epidemics may be supported through burden estimates like ours. Systematic testing of SARI patients can be used to identify the prevalent influenza subtypes and strains that inform vaccine strain selection for in-country influenza vaccines being produced. Our methods largely used existing hospitalization and surveillance data, which will helpful to efforts to replicate or update the results. These and future efforts to better quantify influenza disease burden can be used with vaccine effectiveness and coverage data to estimate potential averted illnesses with vaccination, inform cost-effectiveness analyses, and direct communications to vulnerable populations.

Appendix Additional information about burden of influenza-associated respiratory hospitalizations, Vietnam, 2014–2016.
