# Supplementary material for: Burden of Influenza-Associated Respiratory Hospitalizations, Vietnam, 2014–2016
Source: Emerg Infect Dis. 2021 Oct;27(10):2648–57. doi: 10.3201/eid2710.204765 (PMC8462305; doi:10.3201/eid2710.204765)
Supplement: Appendix — Additional information about burden of influenza-associated respiratory hospitalizations, Vietnam, 2014–2016. [file 20-4765-Techapp-s1.pdf]

Article DOI: https://doi.org/10.3201/eid2710.204765

Burden of Influenza-Associated Respiratory Hospitalizations, Vietnam, 2014–2016

Appendix

Methods for Estimating the Rate of Influenza-Associated Hospitalizations

The influenza-attributable proportion of ARI and SARI hospitalizations was estimated using 2014–2016 SARI sentinel surveillance data. Influenza-associated ARI and SARI hospitalization rates were calculated by multiplying the number of hospitalizations and percent of SARI surveillance patients positive for influenza by age and month, then dividing by census population estimates for each province. The monthly rate estimates were summed to calculate the annual age-adjusted rates as given by the formula:

$$I_a = \sum I_{am} = \frac{\left(H_{am}\frac{F_{am}}{T_{am}}\right)}{P_a}$$

where I_a is the age-specific annual rate of influenza-associated ARI hospitalizations; H_{am} is the age- and month-specific number of hospitalizations; F_{am} is the age- and month-specific number of SARI patients testing positive for influenza; T_{am} is the total number of SARI patients tested for influenza; P_a is the age-specific census population. We calculated the 95% uncertainty intervals (UI) for estimated rates using 1,000 Monte Carlo simulation iterations, assuming a Poisson distribution for the number of hospitalizations and a binomial distribution for the proportion of SARI patients positive for influenza. The same method was used to calculate influenza-associated SARI hospitalization rates, but ARI hospitalization totals were first multiplied by the percent of ARI meeting SARI criteria, given by S_a, to obtain the age- and month-specific number of influenza-associated SARI hospitalizations as given by the adjusted formula:

$$I_a = \sum I_{am} = \frac{\left(H_{am}\left[S_a\frac{F_{am}}{T_{am}}\right]\right)}{P_a}$$

The 95% UIs assumed a binomial distribution for the proportion meeting the SARI case definition. We extrapolated the number of influenza-associated ARI and SARI hospitalizations by multiplying age-specific influenza rates by the provincial census population.

To estimate national rates, age- and month-specific provincial counts of all-cause ARI and SARI and influenza-associated ARI and SARI were each summed across the provinces, and then divided by the total population of the four provinces. The 95% UIs were obtained by calculating the upper and lower 2.5% percentiles from the distribution of provincial rate estimates.

Supplemental Evaluation of Vietnam's Provincial Characteristics Between HAS and Other Provinces

Comparison of provincial characteristics to identify variability between individual provinces and HAS representative provinces within the major health regions of Vietnam.

Objective

To develop a multiplier that adjusts influenza-associated hospitalization rates based on a health statistical score for each province in Vietnam.

Methods

Using data provided in the Health Statistics Yearbook 2015 (Vietnam Ministry of Health's Government Statistics Office, https://thongke.gov.vn/data-and-statistics/2019/10/statistical-yearbook-of-vietnam-2015-2), we evaluated 14 demographic, health and healthcare characteristics potentially associated with variation in hospitalization risks in each province. Each characteristic was calculated as both a percentage and as a normalized value. Two scores were calculated: the average of the percentages and the average of the normalized values. The multiplier for each province was then calculated by dividing the score for each province in a region by the corresponding score for the HAS province in that region (Appendix Table). The range and distribution of average scores and multipliers for each region were summarized.

Covariates

Calculated as percentages and normalized as (value-min/max-min):

% Urban

Physicians per population

Birth rate

Death rate

Under 5 mortality

Literacy

Employment

Income per capita

Health budget

Beds per 100 residents

Nutrition scores: wasting, stunting, underweight

HIV rate

Findings

The average scores varied by a total of only 6 percentage points (average score range = 15.6 – 21.2%). The resulting multiplier therefore ranges from 0.8 – 1.2. The only HAS province that did not have an average score in the middle of the region's range was Quang Ninh; the score was on the high end of the range at 25%, but still within a small score range of 18 – 27% (Appendix Figure 4, 5).

Conclusion

Evaluation by region of 14 demographic, health and healthcare characteristics in each province demonstrated that the provinces in which the HAS was conducted were reasonably representative.

Appendix Table. Multiplier calculation for an example province from each region

Region	Example province	Multiplier calculation
Red River	Hanoi	Average score Hanoi/ Average score Quang Ninh
Northern Mountain	Lao Cai	Average score Lao Cai/Average score Quang Ninh
North Central	Ha Tinh	Average score Ha Tinh/Average score Khanh Hoa
Central Highlands	Gia Lai	Average score Gia Lai/Average score Dak Lak
Mékong River	Ca Mau	Average score Ca Mau/Average score Dong Thap
Southeast	Ho Chi Min City	Average score HCMC/Average score Dong Thap

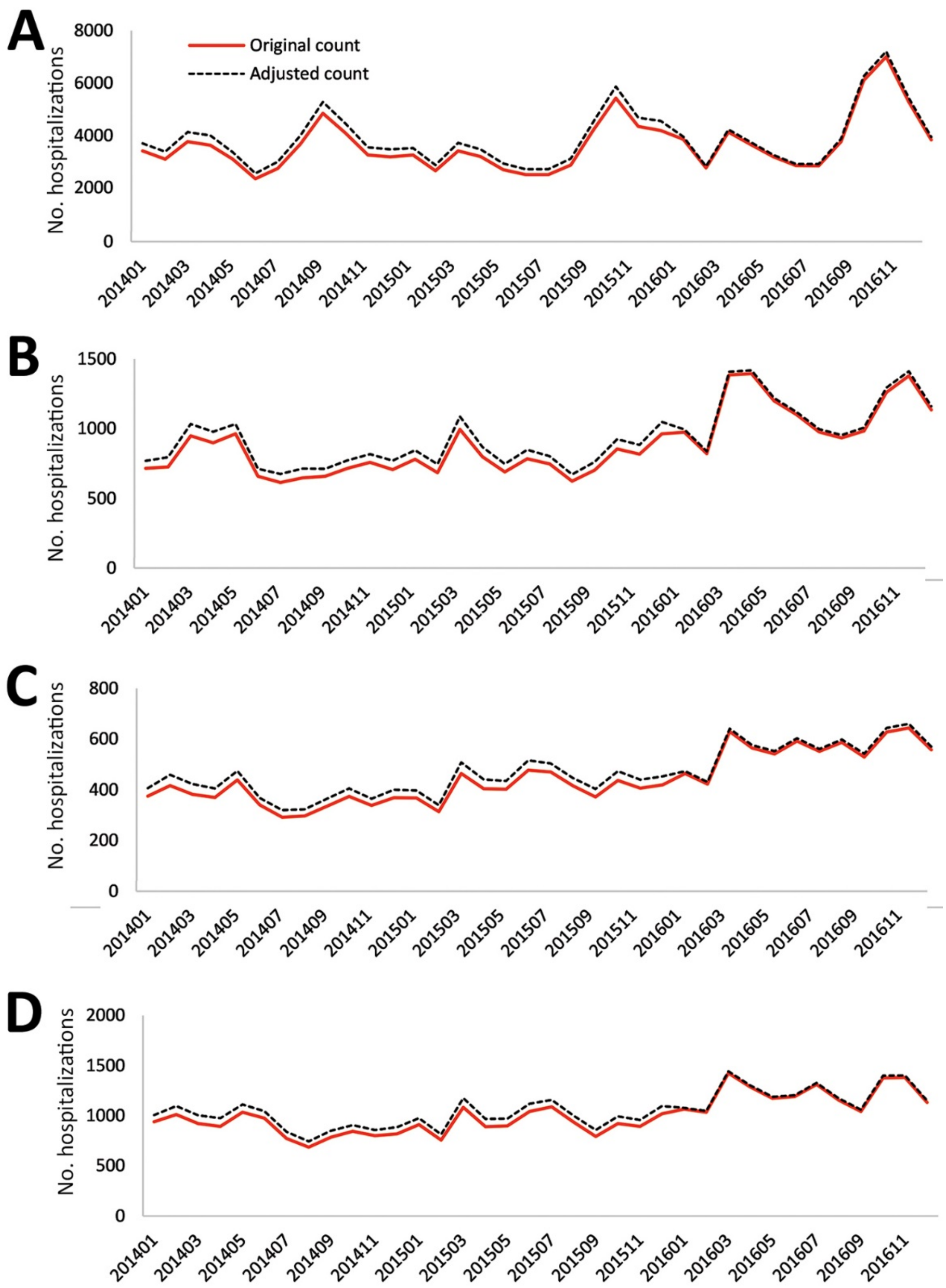

Appendix Figure 1. Comparison of acute respiratory infection hospitalization counts adjusted for hospitals with incomplete electronic medical record implementation in 2014 and 2015. The expected number of ARI hospitalizations for 8 hospitals with clear under-reporting in 2014 and 2015 were calculated using the 2016 percent distribution of counts. The adjusted count reflects a 4.1% adjusted total of 229,144 ARI hospitalizations included in analysis.

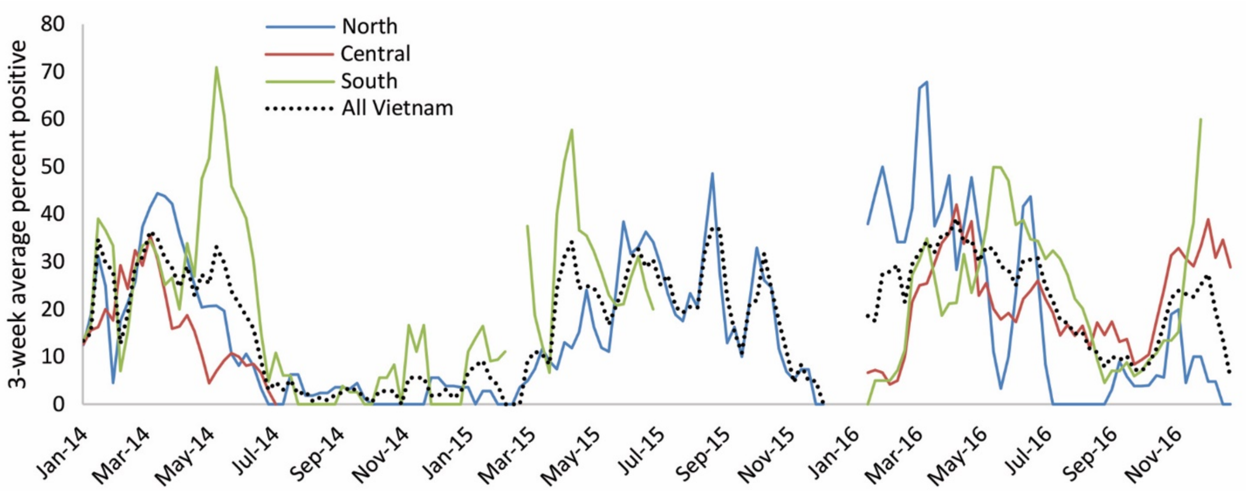

Appendix Figure 2. Comparison of seasonal influenza detection by three major regions of Vietnam, 2014–2016. Central Coast and Central Highland regions were combined due to insufficient data. The weekly percent positive for each region was smoothed using a 3-week moving average of observed data.

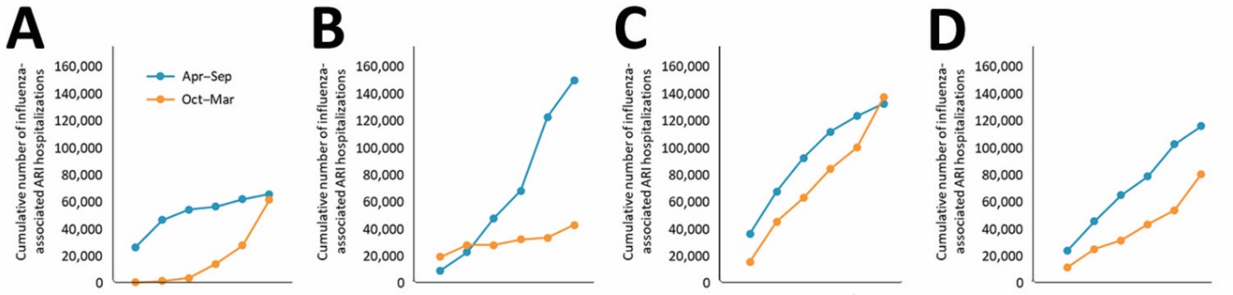

Appendix Figure 3. Cumulative counts of influenza-associated ARI for the 6-month periods following Southern (April to September) and Northern (October to March) Hemisphere influenza vaccination timing 2014–2016 in Vietnam.

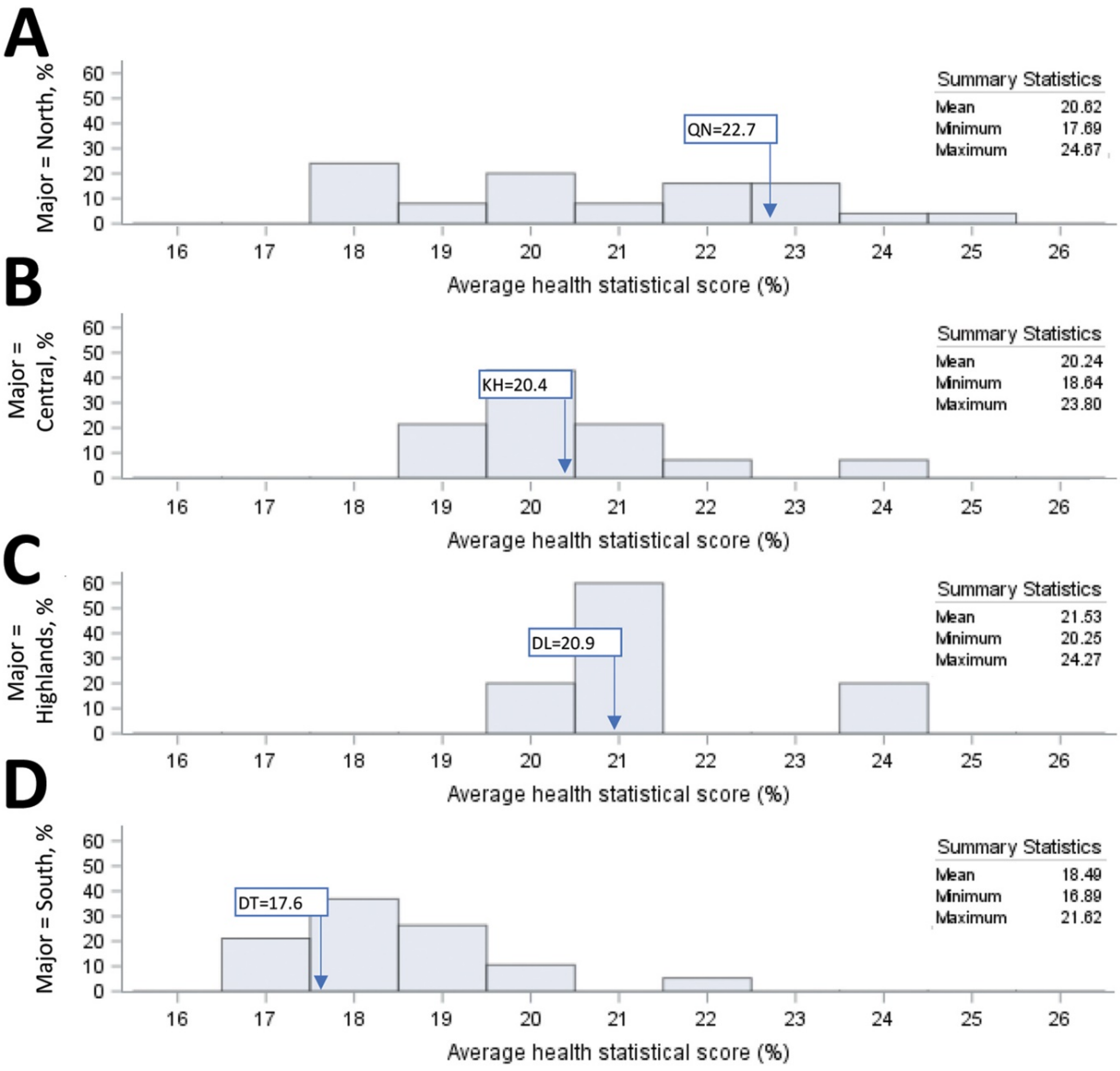

Appendix Figure 4. Health statistical scores calculated from normalized province-level health indicators for each major health region of Vietnam. Inset boxes illustrate the health statistical score of the HAS province representing the region. Abbreviations: Quang Ninh, QN; Khanh Hoa, KH; Dak Lak, DL; Dong Thap, DT. Average scores were calculated from normalized values of 14 different demographic, health and healthcare characteristics potentially associated with variation in hospitalization risks in each province.

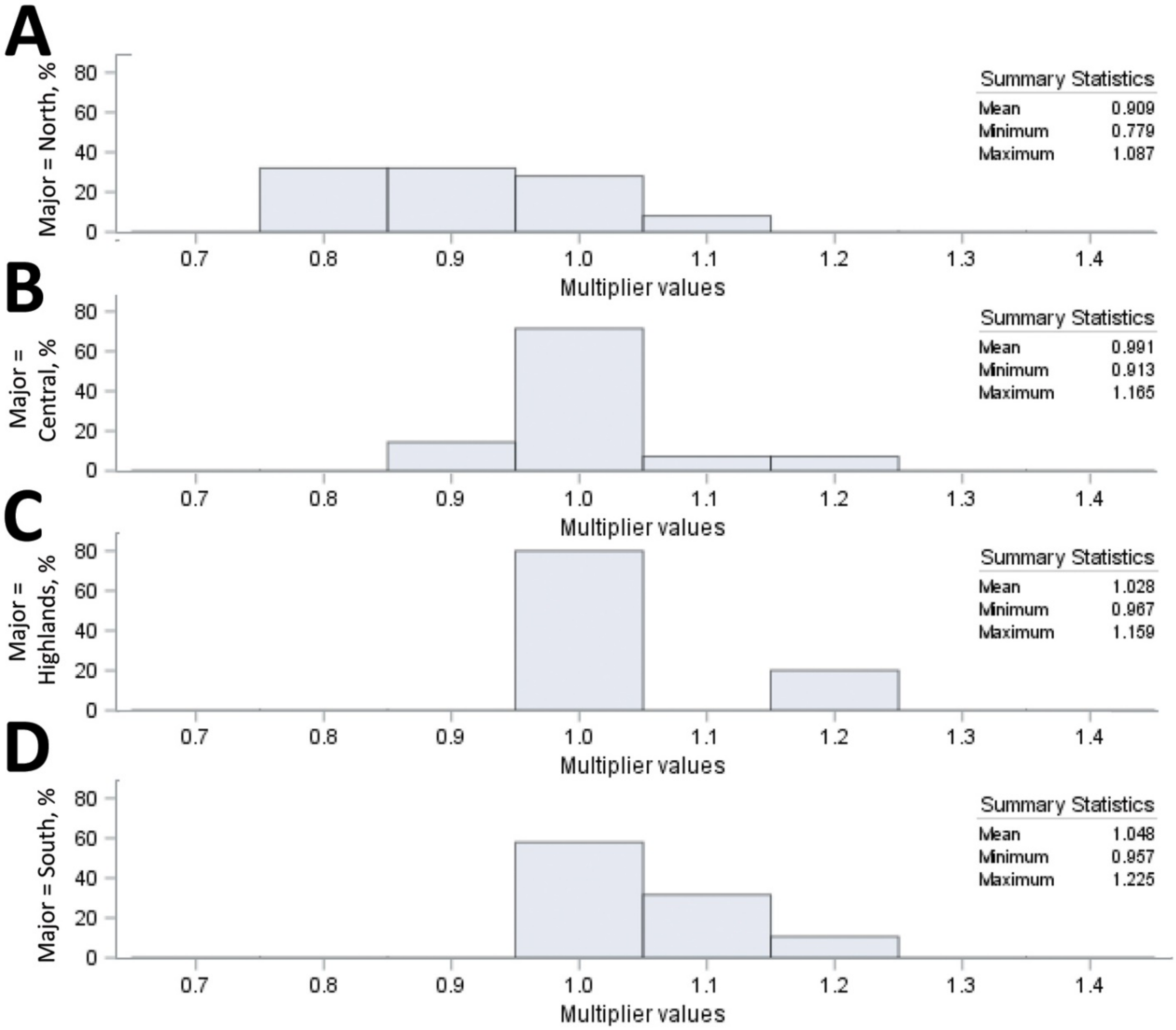

Appendix Figure 5. Demonstration of minimal variation in the range and distribution of average health statistical scores for each major health region of Vietnam. Multiplier values were calculated by dividing the score for each province in a region by the corresponding score for the HAS province in that region.
